# Diels–Alder Polymer Networks with Temperature‐Reversible Cross‐Linking‐Induced Emission

**DOI:** 10.1002/anie.202013183

**Published:** 2020-11-19

**Authors:** Yu Jiang, Nikos Hadjichristidis

**Affiliations:** ^1^ Key Laboratory of Catalysis and Energy Materials Chemistry of Ministry of Education & Hubei Key Laboratory of Catalysis and Materials Science Hubei R&D Center of Hyperbranched Polymers Synthesis and Applications South-Central University for Nationalities Wuhan 430074 China; ^2^ Polymer Synthesis Laboratory KAUST Catalysis Center Physical Sciences and Engineering Division King Abdullah University of Science and Technology (KAUST) Thuwal 23955 Saudi Arabia

**Keywords:** cross-linking-induced emission, Diels–Alder reaction, polymers, reversible photoluminescence, temperature response

## Abstract

A novel synthetic strategy gives reversible cross‐linked polymeric materials with tunable fluorescence properties. Dimaleimide‐substituted tetraphenylethene (TPE‐2MI), which is non‐emissive owing to the photo‐induced electron transfer (PET) between maleimide (MI) and tetraphenylethene (TPE) groups, was used to cross‐link random copolymers of methyl (MM), decyl (DM) or lauryl (LM) methacrylate with furfuryl methacrylate (FM). The mixture of copolymer and TPE‐2MI in DMF showed reversible fluorescence with “on/off” behavior depending on the Diels–Alder (DA)/retro‐DA process, which is easily adjusted by temperature. At high temperatures, the retro‐DA reaction is dominant, and the fluorescence is quenched by the photo‐induced electron transfer (PET) mechanism. In contrast, at low temperatures, the emission recovers as the DA reaction takes over. A transparent PMFM/TPE‐2MI polymer film was prepared which shows an accurate response to the external temperature and exhibited tunable fluorescent “turn on/off” behavior. These results suggest the possible application in areas including information security and transmission. An example of invisible/visible writing is given.

## Graphical abstract

Novel Diels–Alder polymer networks with tunable aggregation‐induced emission (AIE) behavior, constructed from non‐emission dimaleimide‐substituted tetraphenylethene and linear furan‐based copolymers, have a tunable fluorescent “turn on/off” property, which shows an accurate response to temperature.

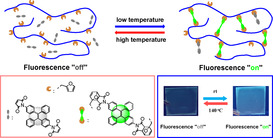

## Introduction

Covalently cross‐linked materials (thermosets) have played an important role in many areas such as coatings, adhesives, composite materials, and biomaterials. They have shown outstanding properties (e.g., modulus, thermostability, etc.) compared to the linear thermoplastics.^[1]^ However, permanently covalent cross‐links also bring disadvantages for the processing, recycling, and reuse of these materials.^[2]^ To overcome these barriers, reversible covalent bonds (dynamic or non‐dynamic) were introduced to construct the reversible cross‐linked materials by using a variety of chemistries, such as disulfide metathesis,^[3]^ transesterification,^[4]^ transamination,^[5]^ Diels–Alder (DA) reaction,^[6]^ and many more.^[7]^ Among these chemistries, thermal‐induced DA reaction is one of the best options due to its catalyst‐free procedure, broad temperature range, and high tolerance.^[8]^ As a result, the chain topology can be adjusted by the formation or rupture of cross‐link bonds. This approach ensures the tailorable properties of reversibly cross‐linked materials, which behave as thermosets or thermoplastic materials depending on the formation or fracture of the cross‐links.^[9]^ A plethora of efforts has been made to expand the application of reversibly cross‐linked materials in many areas,^[10]^ while there are very few in the field of reversible/tunable fluorescence.

Luminescent materials have attracted considerable attention in recent decades, as they have promising applications in luminescent sensing, information encoding, and biological imaging.^[10]^ The application of conventional organic luminogens was limited by the aggregation‐caused quenching (ACQ) effect, which means that the fluorescence of luminogens undergoes complete quenching in the aggregation state.^[11]^ Recently, Tang's group has developed a novel aggregation‐induced emission (AIE)‐type luminogens, which show the opposite phenomenon of ACQ emitting strongly in the aggregation state.^[12]^ By introducing the AIE‐gens into the polymer structures, the resulted polymer possesses the polymer properties as well as the AIE characteristics.^[13]^ These polymers show many advantages over the small molecules, such as processability, good thermal stability, structural diversity, and regulation of AIE behavior.^[14]^ Actually, there are many studies that have been reported to regulate the fluorescence of AIEgen‐containing polymers by changing the aggregation state,^[15]^ self‐assemble behavior,^[16]^ topology,^[17]^ etc.^[18]^ However, for cross‐linked polymeric materials, it is difficult to regulate their emission behavior due to the insoluble and infusible nature. The development of cross‐linked materials with a reversibly covalent bond is helpful to solve this problem. Ji's group recently reported that AIE luminogens‐doped vitrimers could exhibit tunable fluorescence depending on the movement of the network at different temperature.^[19]^ Herein, we wish to report the preparation of reversibly DA polymer networks in which the AIEgens are incorporated into the network. The fluorescence property can be regulated by the cross‐linking/decross‐linking process through DA/retro‐DA chemistry. Most importantly, the emission behavior of the resulted materials exhibits an alterable “turn‐on/off” mode.

**Scheme 1:**
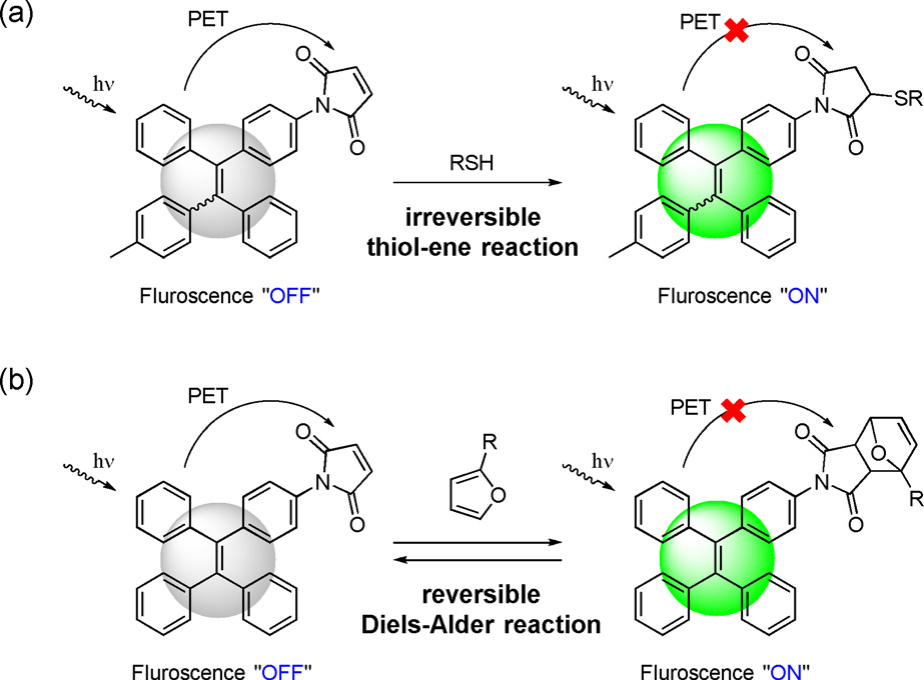
a) Fluorescent “turn on” strategy with irreversible thio‐ene reaction based on photo‐induced electron transfer (PET) process; b) Fluorescent “turn on/off” strategy with reversible DA reaction in the present work.

**Scheme 2:**
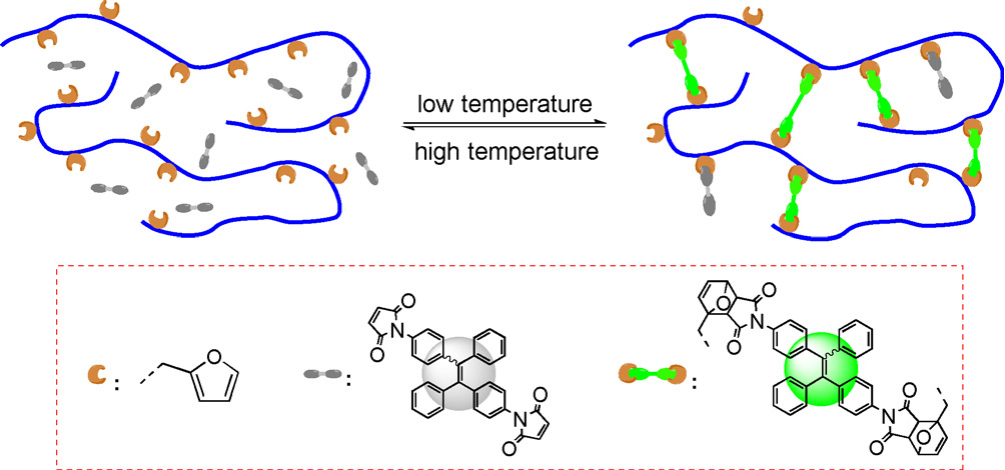
Temperature‐reversible cross‐linking‐induced emission using a DA/retro‐DA strategy.

Photo‐induced electron transfer (PET) has been widely utilized as a mechanism for the development of fluorescent “turn‐on” materials.^[20]^ “Fluorophore‐spacer‐receptor” format was usually adopted as the fluorescent PET‐based system. For example, the maleimide (MI) group was introduced into the tetraphenylethene (TPE) molecule, and the resulting TPE‐MI showed no emission in both solution and solid‐state, because the intramolecular PET process between fluorophore (TPE) and receptor (MI) quench the fluorescence. When the MI group is bonded by a thiol‐ene reaction, the fluorophore (TPE) lights up because the electron transfer process is over (Scheme 1 a).^[21]^ Compared to the irreversible thio‐ene reaction, the reversible DA reaction between MI and the furan group should result in the tunable fluorescence of the adduct (Scheme 1 b).^[22]^ In the present work, we incorporate, for the first time, MI‐substituted AIE‐gens into the polymer networks by using dimaleimide‐substituted tetraphenylethene (TPE‐2MI) as a cross‐linker (Scheme 2). The breakage of PET process by reaction of TPE‐2MI with furan moieties releases the AIE behavior, and the cross‐linked networks restricts the mobility of TPE molecules to enhance their emission. This phenomenon, of the reversible cross‐linking‐induced‐emission, has not been reported so far. By using this method, it is possible to regulate the emission of cross‐linked materials by chemistry. Besides, the relationship between emission and cross‐linking also indicates a potential application for the visualization of the dynamic cross‐linking process. The resulted networks exhibit reversible emission behavior thanks to the temperature‐responsive DA/retro‐DA reaction.

## Results and Discussion

**Scheme 3:**
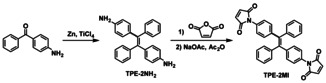
Synthesis of TPE‐2MI.

**Scheme 4:**
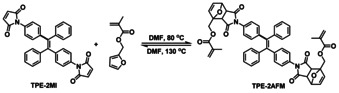
The model reaction of TPE‐2MI with furfuryl methacrylate.

As illustrated in Scheme 3, TPE‐2MI was synthesized from 4‐aminobenzophenone by the McMurry coupling reaction in the first step, followed by transformation of the ‐NH_2_ to the maleimide group. The chemical structure of TPE‐2MI was confirmed by ^1^H and ^13^C NMR (Figure S1, SI). Then the model reaction between TPE‐2MI and furfuryl methacrylate (FM) was performed in DMF at 80 °C to check the reactivity of the reversible DA reaction and the AIE property of DA adduct TPE‐2AFM (Scheme 4). The TPE‐2AFM adduct was characterized by ^1^H NMR, as shown in Figure S2a (SI). It is well known that the DA reaction leads to a mixture of two diastereomers (*endo*/*exo* adducts), and both of them are found in our ^1^H NMR spectrum. Comparison with that of TPE‐2MI (Figure S1a, SI), reveals that the resonance signal at *δ* 6.83 belonging to maleimide group (d and e) disappeared and new signals d′ and e′ (*endo* adduct), d′′ and e′′ (*exo* adduct) between 3.00–3.80 ppm appeared, thus proving the success of DA reaction. Moreover, the TPE‐2AFM undergoes a retro‐DA reaction entirely within 5 min at a high temperature (Scheme 4). The entire disappearance of signals of TPE‐2AFM from ^1^H NMR spectrum proved the success of the retro‐DA reaction. The obtained TPE‐2MI and furfuryl methacrylate were also confirmed by the ^1^H NMR spectrum (Figure S2b, SI). These results show efficient DA and retro‐DA reaction between TPE‐2MI and furfuryl methacrylate under different temperatures.

**Figure 1:**
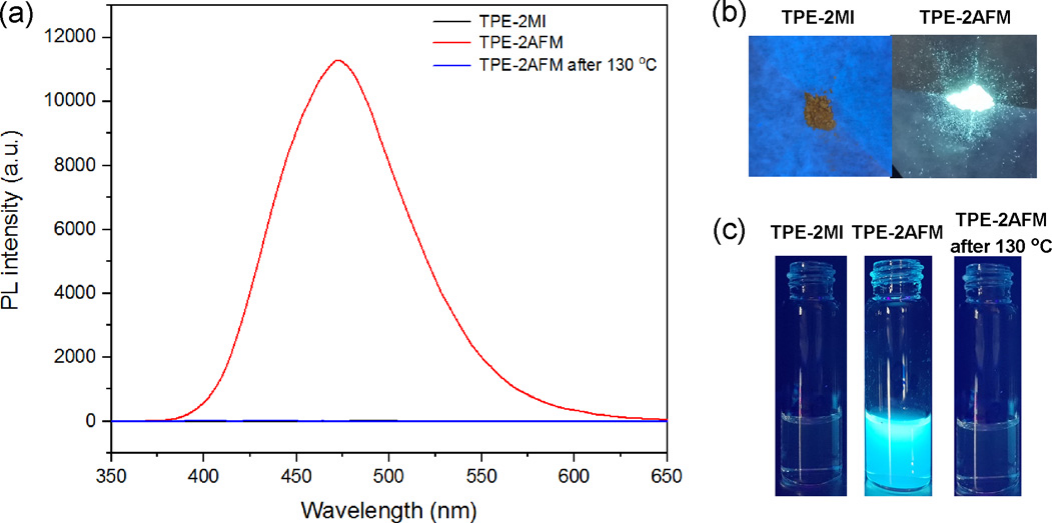
a) Photoluminescence (PL) spectra of TPE‐2MI, TPE‐2AFM, and retro‐DA product in THF/H_2_O mixtures (80 % H_2_O) at a concentration of 0.1 g L^−1^ (excitation: 342 nm, temperature: 25 °C); b) Photos of TPE‐2MI and TPE‐2AFM solid powders taken under 365 nm UV irradiation; c) Photos of TPE‐2MI, TPE‐2AFM, and retro‐DA product in THF/H_2_O mixtures (80 % H_2_O) at a concentration of 0.1 g L^−1^ under 365 nm UV irradiation.

The emission characteristics of TPE‐2MI and TPE‐2AFM were then investigated. TPE‐2MI was non‐emissive in either solution or solid‐state, while the TPE‐2AFM exhibited strong emission under UV light (Figure 1 b,c and Figure S5). Furthermore, the AIE behavior of TPE‐2AFM was confirmed by the investigation of its THF/H_2_O solutions, and the results were shown in Figure S6 (SI). The addition of H_2_O into the THF solution induced the molecular aggregation (confirmed by dynamic light scattering measurements) and gradually enhanced its photoluminescence (PL) intensity. The maleimide groups, which act as potent quenchers of TPE fluorogens via a PET mechanism in TPE‐2MI, are bonded in TPE‐2AFM through the DA reaction. This results in the strong emission of TPE‐2AFM. After retro‐DA reaction, the maleimide groups are released, and PET works again to quench significantly the fluorescence (Figure 1 a and c). This model study indicates that the fluorescent “turn on/off” behavior is regulated by the temperature‐responsive DA/retro‐DA reaction.

**Scheme 5:**
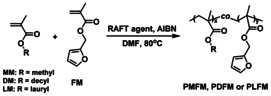
Synthesis of a random copolymer of methyl methacrylate (MM), decyl methacrylate (DM), or lauryl methacrylate (LM) with furfuryl methacrylate (FM) by the reversible addition‐fragmentation chain transfer polymerization.

With the successful results of the model study in hand, the Diels–Alder reversible polymer networks were prepared from TPE‐2MI and a random copolymer of methyl/decyl/lauryl methacrylate and furfuryl methacrylate (PMFM/PDFM/PLFM). Four random copolymers with different composition and molecular weight were synthesized by the reversible addition‐fragmentation chain transfer polymerization (RAFT) with AIBN as initiator and 2‐cyano‐2‐propyl dodecyl trithiocarbonate as a chain‐transfer agent (Scheme 5). The molecular weight, determined by ^1^H NMR and the polydispersity index Đ by gel permeation chromatography (GPC) with polystyrene standards, are given in Table 1. The calculated composition of the random copolymers is consistent with the feed ratios of the two monomers (Figure S3, SI). All GPC traces are monomodal with narrow molecular weight distributions (Figure S11,12, SI).

**Table 1:** Molecular characteristics of the random copolymers PMFM, PDFM, and PLFM.

Entry	Sample	*M*_n, NMR_^[a]^	DP^[a]^		*Đ* ^[b]^
			Co‐monomer	FM	
1	PM_62_F_24_M	10 500	62	24	1.09
2	PM_60_F_40_M	13 000	60	40	1.13
3	PD_60_F_20_M	18 500	60	20	1.25
4	PL_100_F_10_M	27 000	100	10	1.45

[a] *M*_n, NMR,_ and degree of polymerization (DP) were calculated from ^1^H NMR spectra (400 MHz, CDCl_3_, 25 °C); [b] *Đ* (*M*_w_/*M*_n_) was determined by GPC (THF, 25 °C, PS standards).

The Diels–Alder polymer networks were prepared by dissolving the linear copolymer and cross‐linker TPE‐2MI (maleimide unit/furan unit ratio, *I*_mal/fur_=1) in DMF. In principle, the furan and maleimide groups undergo DA reaction below a specific temperature (usually 80 °C). At higher temperatures (≥130 °C), furan‐maleimide linkages will cleave through retro‐DA reaction to form the initial linear copolymer chain materials. To gain an in‐depth insight into the cross‐linking/uncross‐linking process, a series of experiments were performed. Firstly, ^1^H NMR spectroscopy was used to check the DA adduct formation. A mixture of PM_60_F_40_M and TPE‐2MI (maleimide unit/furan unit ratio, *I*_mal/fur_=1) in [D_7_]DMF was allowed to react at 80 °C, and the ^1^H NMR spectra were collected at different time intervals (Figure S4, SI). After 1.5 h, new signals around *δ* 4.65, 4.95, 5.51 ppm appeared, which are similar to the chemical shift of signals of the TPE‐2AFM spectrum (Figure S2 a). The increase of integration of these peaks indicated the successful DA reaction. The solution cannot be monitored after 5 h due to its high viscosity. Then the mixture was heated at 140 °C for 5 min, the viscosity decreased, and all the aforementioned signals disappeared, suggesting the progress of retro‐DA reaction. Heat‐induced chemical cross‐linking/uncross‐linking process was further confirmed by using differential scanning calorimetry (DSC). DMF solution of PM_60_F_40_M/TPE‐2MI (*I*_mal/fur_=1) was heated to 80 °C, and small volumes of the DMF solution were taken out at different time intervals for characterization. The cross‐linked networks were obtained after removing the DMF and unreacted small molecular. As showing in Figure S13 (SI), PM_60_F_40_M has a distinct glass transition temperature (*T*_g_) at 83.5 °C. During the cross‐linking process, the *T*_g_ of the cross‐linked networks increased over time, which reached its plateau value at 109.5 °C after 24 h. This could be ascribed to the cross‐linking induced constraints of polymer chains. Comparison to that of linear PM_60_F_40_M, the *T*_g_ of the formed networks become broad due to the heterogeneous dynamics. Moreover, the DSC of networks also showed two endothermic peaks at the temperature around 140 and 160 °C, indicating the retro‐DA reaction of *endo* and *exo* adduct (Figure S14, red line, SI). All of these results prove the formation of cross‐linked polymer networks with DA reaction at lower temperatures and its uncross‐linking process at higher temperatures.

**Figure 2:**
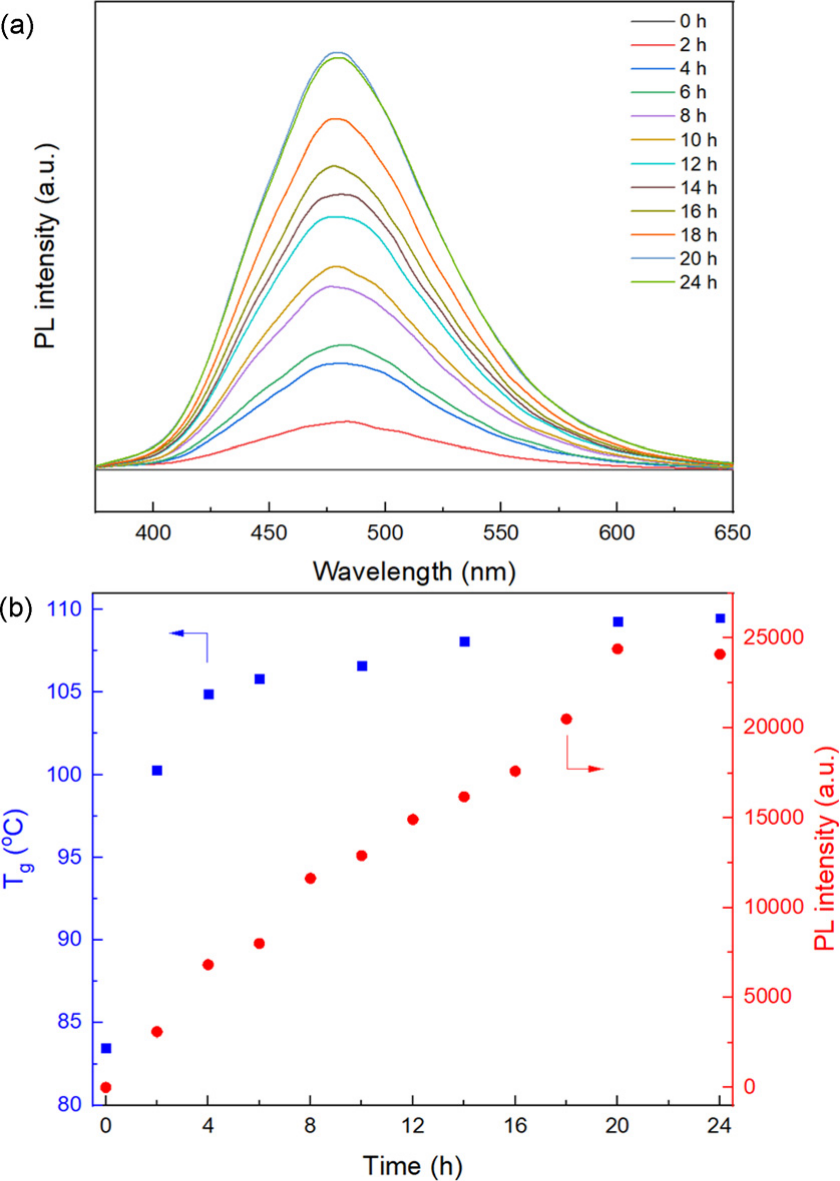
a) PL spectra of mixture of PM_60_F_40_M/TPE‐2MI in DMF (concentration: 0.8 g L^−1^) at different time intervals (excitation: 342 nm, temperature: 25 °C); b) *T*_g_ (blue) of cross‐linked networks and PL intensity (red) at 483 nm versus reaction time at 80 °C.

The fluorescence of the polymer networks was then studied. As the DA reaction proceeds, the viscosity of the DMF solution increased. The cross‐linked polymer was swelling, and the DMF solution was taken out and diluted to monitored the PL intensity. The unreacted mixture PM_60_F_40_M/TPE‐2MI showed no emission due to the PET effect between maleimide and TPE groups, but as the DA reaction goes on, it becomes fluorescent. As shown in Figure 2 a, the PL intensity of the solution is gradually enhanced with increasing reaction time and reaches its highest value after approximately 20 h. It shows a similar tendency with *T*_g_ value, which rises before 20 h and reaches its highest value after that (Figure 2 b). These results indicate that the PL intensity increases along with the degree of cross‐linking. The PM_60_F_40_M/TPE‐2MI mixture achieves an equilibrium state around 20 h at 80 °C, and the *T*_g_ and PL intensity do not increase anymore after that. This phenomenon indicates that the dynamic cross‐linking process can be visualized by monitoring the PL intensity.

**Figure 3:**
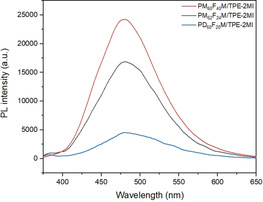
PL spectra of DMF solution of PMFM/TPE‐2MI or PDFM/TPE‐2MI after heating at 80 °C for 20 h (excitation: 342 nm, temperature: 25 °C).

The effects of the composition of random copolymers and the FM comonomer (MM or DM) on PL intensity were also investigated. For this purpose, the other two random copolymers PM_62_F_24_M and PD_60_F_20_M were used to form networks with TPE‐2MI in DMF at 80 °C for 24 h. The influence of the furan density along the chain on the PL intensity can be elucidated by comparing samples 1 and 2 (Table 1). On one hand, the influence of the presence of FM comonomers on the PL can be found by comparing samples 1 and 3 (Table 1). In all cases, the molecular ratio was maintained at *I*_mal/fur_=1. Besides, all results were normalized to correspond to the same DP. It is clear that by increasing the furan group density, the cross‐linking density is increased and, consequently, the PL intensity (Figure 3). On the other hand, the PD_60_F_20_M/TPE‐2MI reaches its equilibrium faster than PM_62_F_24_M/TPE‐2MI (Figure S8, SI), which indicates a lower cross‐linking density due to the long pendant decyl group. As a result, it shows lower PL intensity compared to that of the PM_62_F_24_M/TPE‐2MI (Figure 3).

**Figure 4:**
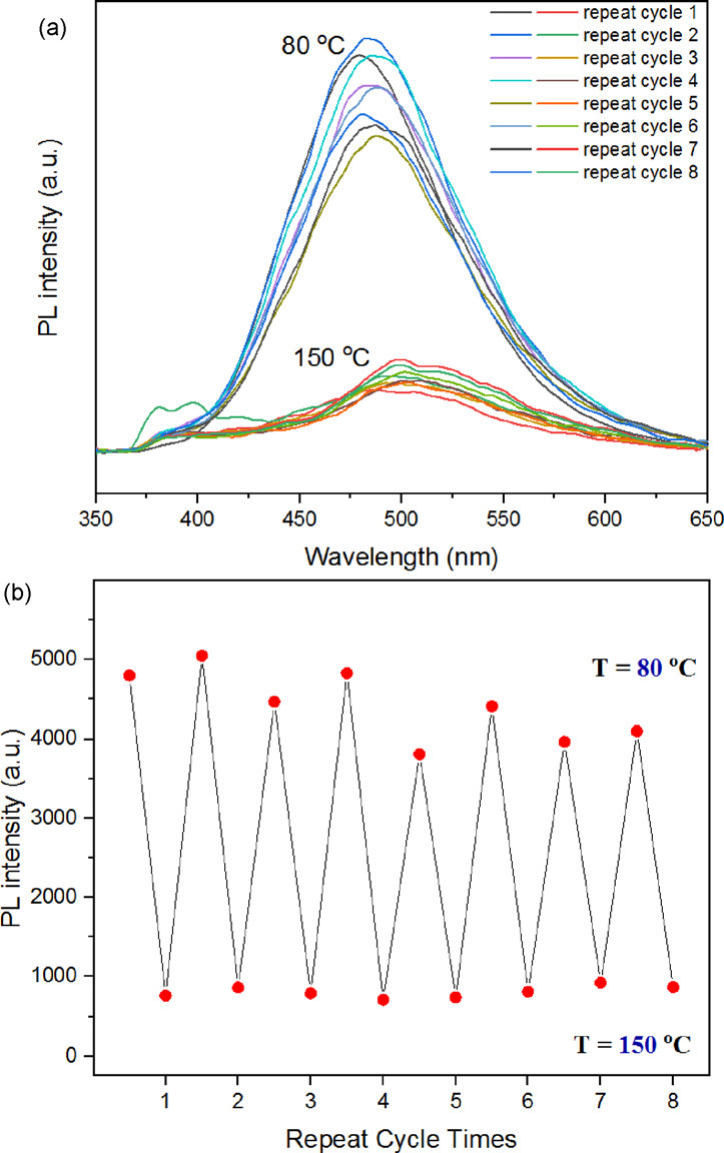
PL spectra (a) and peak intensity at 483 nm (b) of PM_60_F_40_M/TPE‐2MI networks in DMF solution (concentration: 0.8 g L^−1^) for several cycles by adjusting temperature between 80 °C (4 h) and 150 °C (10 min); (excitation: 342 nm, temperature: 25 °C).

The temperature‐responsive DA/retro‐DA reaction has played a crucial role in the emission of cross‐linked networks. For the cross‐linked polymers, at a lower temperature (≤80 °C), the maleimide group of TPE‐2MI reacts with the furan group, and the PET effect disappeared. Once the temperature is above 130 °C, the retro‐DA reaction is dominant. The released maleimide groups make fluorescence to be quenched again. For instance, the emission of the DMF solution of PM_60_F_40_M/TPE‐2MI reached its highest intensity after 20 h at 80 °C, and then disappeared in 10 min when the temperature was further increased to 150 °C. The fluorescent intensity was weakened rapidly with the rise of temperature due to the change of PET effect in the TPE‐2MI unit. The results of the reversible cycle experiment are shown in Figure 4. The cross‐linked networks exhibited reversible fluorescent “turn on/off” behavior, which was easily regulated by adjusting temperature between 80 and 150 °C. The broad peak around 360–440 nm in the spectrum of cycle 8 is due to impurities in DMF; see Figure S7 in SI.

To further expand the potential application of this strategy, the construction of reversibly cross‐linked networks in solvent‐free conditions was investigated. PM_60_F_40_M and TPE‐2MI (*I*_mal/fur_=1) were dissolved in dichloromethane, followed by immediate removal of the solvent under vacuum and curing at room temperature. The *T*_g_ of the bulk PM_60_F_40_M/TPE‐2MI increased over time and reached a plateau value of around 95.2 °C after 1 week (Figure S14, blue line, SI). Comparing to that of the cross‐linking process in DMF at 80 °C, the *T*_g_ value was lower, indicating the lower degree of cross‐linking. And only one endothermic peak at a temperature around 140 °C was found, indicating that only *endo* adduct was formed at room temperature. The Diels–Alder reaction rate can also be accelerated by increasing the temperature in solid‐state, and the ratio of the *exo* adduct will increase along with the increasing temperature. The fluorescence of bulk PM_60_F_40_M/TPE‐2MI mixture also showed reversible responsiveness to different temperatures. As shown in Figure S19 (SI), the PM_60_F_40_M/TPE‐2MI mixture in a flask shows no emission under UV light at 0 h (immediately after solvent removal). It emits a strong fluorescence after few hours illustrating the formation of polymer networks. Then the lower half of the flask was put into an oil bath at 140 °C for 10 mins, and an interesting phenomenon was observed. The sample at the lower half area of the flask showed non‐emission, while the sample at the upper half area was strongly emitting. This phenomenon indicates that the emission of cross‐linked polymeric materials has a fast and accurate response to high temperatures.

Due to their high *T*_g_, both cross‐linked polymers PMFM/TPE‐2MI (*T*_g_=109 °C) and PDFM/TPE‐2MI (*T*_g_=69 °C) are vitrified at room temperature. Therefore, it is necessary to investigate if their emission is caused by vitrification, as in the TPE‐doped polymers,^[23]^ instead of cross‐linking. For this purpose, a network with low *T*_g_ (−55 °C, Figure S16, SI) and low cross‐linking density was prepared from a flexible linear copolymer PL_100_F_10_M (Table 1) and TPE‐2MI. The bulk PL_100_F_10_M/TPE‐2MI mixtures (*I*_mal/fur_=1 or 0.5) become insoluble in DMF at room temperature, which indicates the successful cross‐linking process (Figure S18, SI). The endothermic peak at the temperature around 110 °C in DSC traces also suggested the retro‐DA reaction of the *endo* adduct (Figure S16, SI). As shown in Figure S16, the *T*_g_ of the bulk PL_100_F_10_M/TPE‐2MI has no obvious change during the cross‐linking process, probably due to the long lauryl chain and low cross‐linking density (Figure S16). These low *T*_g_ values indicate that the obtained polymer networks are not vitrified at room temperature. The fluorescence property of the bulk PL_100_F_10_M/TPE‐2MI was also monitored. As shown in Figure S9, the PL intensity increased gradually as the cross‐linking proceeded, despite the networks were not vitrified. These results indicate that the emission of the networks is caused by cross‐linking instead of vitrification. In order to further support this conclusion, another TPE‐doped cross‐linked polymer was prepared by mixing PL_100_F_10_M, 1,1′‐(methylenedi‐4,1‐phenylene)bismaleimide (BMI) and TPE in dichloromethane (*I*_mal/fur_=0.5), followed by immediate removal of the solvent under vacuum and curing at room temperature. BMI, one of the most commonly used linker in DA cross‐linked polymers, was used to cross‐link linear copolymer PL_100_F_10_M to form the polymer networks with low *T*_g_ (Figure S17, SI). However, this bulk PL_100_F_10_M/BMI/TPE mixture was non‐emissive either before or after cross‐linking (Figure S10, SI). The movement of the polymer chains greatly increases the freedom for TPE and therefore its rotation intramolecularly. While for TPE‐2MI cross‐linked polymers, the TPE molecules are integrated into the rigid network, which restrict the intramolecular rotation of TPE molecules. Although the networks are not vitrified at room temperature, the movement of the long lauryl chain can rarely influence the mobility of TPE molecules which are incorporated in the network. In other words, the formed networks can sufficiently restrict the rotation of the TPE molecules, no matter whether the cross‐linked polymers are vitrified or not. As a comparison, the cross‐linking density, which can directly influence the stiffness of the network, has more significant influence on the rotation of TPE molecules, thus influence the fluorescence property (as the comparison between PD_60_F_20_M/TPE‐2MI and PM_62_F_24_M/TPE‐2MI). It can be concluded that the increase of the PL intensity during the cross‐linking process depends on the following two factors: a) the continuous DA reaction, which breaks the PET process between TPE and MI groups, and b) the restricted mobility of the chains due to the formation of the network, which greatly restricts the intramolecular rotation of TPE.

**Figure 5:**
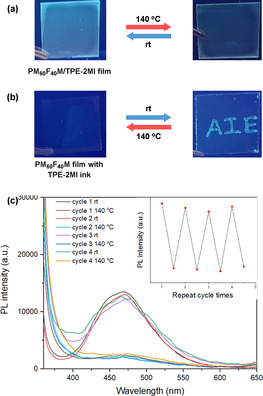
a) Photos show the fluorescent “turn on/off” cycle under 365 nm UV light; b) Photos show the “writing–erasing” cycle under 365 nm UV light; c) PL spectra and peak intensity at 483 nm (inset) of PM_60_F_40_M/TPE‐2MI mixture film for several cycles by adjusting temperature between room temperature (24 h) and 140 °C (10 min).

We further performed fluorescence turn “on/off” experiments to show the potential applications of this strategy, such as temporary information transmission. A transparent PM_60_F_40_M/TPE‐2MI film was then prepared by the spin coating process on a glass substrate, and its reversible emission performance was studied (Figure 5 c). The emission of polymer film exhibited “turn on/off” behavior depending on the environment temperature (Figure 5 a), and the process can be repeated a few times. Furthermore, the TPE‐2MI solution was further used as an ink to write on the PM_60_F_40_M coated surface. As illustrated in Figure 5 b, the “AIE” letters could be written on the PM_60_F_40_M film, which is invisible under UV light at the beginning, but they appear after few hours as the networks formed. The emission intensity became stronger over time. Moreover, the “AIE” letters could be rapidly erased in 10 mins by increasing the environment temperature to 140 °C, and it can be recovered in a few hours if the high‐temperature environment was removed.

## Conclusion

In summary, we have synthesized a non‐emitting TPE‐2MI that can be used as a cross‐linker for furan‐based random copolymers to construct polymer networks through Diels–Alder strategy. The PL intensity of the polymer networks increases with the cross‐linking density, which indicates that this strategy can be used to visualize the dynamic cross‐linking process. The resulted networks show strong emission due to the break of the PET process between TPE and MI groups, as well as the restricted intramolecular rotation of the TPE molecules. The PL intensity of the networks can be enhanced by increasing the density of the furan unit and shortening the pendant chain of the linear copolymer because both parameters are increasing the cross‐linking density. Moreover, a novel reversible covalently cross‐linked polymeric material with tunable fluorescence was obtained. The polymer networks in DMF shows tunable fluorescence property depending on the cross‐linking/uncross‐linking process. With the increase of temperature (≥130 °C), the formed polymer networks undergo retro‐DA reaction, and the fluorescence is quenched. On the contrary, the emission is recovered at low temperature (≤80 °C) due to the DA reaction and suspension of the PET mechanism. This behavior with “turn on/off” mode can be easily regulated by temperature. Furthermore, a transparent PMFM/TPE‐2MI film was prepared, and its emission also is shown “turn on/off” behavior, with a fast and accurate response to the environment temperature. As an application example, the successful “writing‐erasing” on a polymer surface indicates the potential application of this material in many areas, such as temporary information transmission.

## Conflict of interest

The authors declare no conflict of interest.

## Supporting information

As a service to our authors and readers, this journal provides supporting information supplied by the authors. Such materials are peer reviewed and may be re‐organized for online delivery, but are not copy‐edited or typeset. Technical support issues arising from supporting information (other than missing files) should be addressed to the authors.

Supplementary
