# Supplementary material for: Diels–Alder Polymer Networks with Temperature‐Reversible Cross‐Linking‐Induced Emission
Source: Angew Chem Int Ed Engl. 2020 Nov 19;60(1):331–7. doi: 10.1002/anie.202013183 (PMC7839674; doi:10.1002/anie.202013183)
Supplement: Supplementary file 1 — Supplementary [file ANIE-60-331-s001.pdf]

Angewandte Chemie
Eine Zeitschrift der Gesellschaft Deutscher Chemiker

Supporting Information

# Diels–Alder Polymer Networks with Temperature-Reversible Cross-Linking-Induced Emission

*Yu Jiang* and Nikos Hadjichristidis**

anie_202013183_sm_miscellaneous_information.pdf

# Supporting Information

## 1. EXPERIMENTAL SECTION

**Materials.** 4-aminobenzophenone (98%), zinc powder, titanium tetrachloride ($TiCl_4$, ≥ 98%), potassium carbonate (anhydrous, ≥ 99%), sodium acetate (anhydrous, ≥ 99%), acetic anhydride (99%), maleic anhydride (≥ 99%), 2,2'-azobis(isobutyronitrile) (AIBN, 99%), 2-cyano-2-propyl dodecyl trithiocarbonate (97%), 1,1'-(methylenedi-4,1-phenylene)bismaleimide (BMI, 95%) tetraphenylethene (TPE, 98%) were purchased from Aldrich and used as received. *N, N*-dimethylformamide (DMF, 99%), furfuryl methacrylate (97%), n-lauryl methacrylate (96%), decyl methacrylate (≥ 99%), and methyl methacrylate (≥ 99%) were distilled over calcium hydride ($CaH_2$) before use. Tetrahydrofuran (THF) was refluxed over sodium/benzophenone and distilled under a nitrogen atmosphere just before use.

**Measurements.** The gel permeation chromatography (GPC) measurements of PMFM and PDFM were carried out with the VISCOTEK VE2001 equipped with Styragel HR2 THF and Styragel HR4 THF columns using THF (1mL/min) as eluent. The gel permeation chromatography (GPC) measurement of PLFM was carried out with Agilent HPLC system equipped with One PLgel 5 μm guard column and three Mz-Gel $SD_{plus}$ columns using THF (1mL/min) as eluent. Both systems were calibrated with linear polystyrene. The NMR spectra were recorded with a Bruker AVANCE III-400 or 500 spectrometers. Differential scanning calorimetry (DSC) measurements were performed using a Mettler Toledo DSC1/TC100 system under an inert atmosphere (nitrogen). Photoluminescence spectra were recorded on a Thermo Lumina Fluorescence Spectrometer equipped with an external water circulator for the thermostatted cell holder. Dynamic light scattering (DLS) measurements were carried out with a Malvern Zetasizer Nano ZS device equipped with a TurboCorr correlator. The light source was a 30 mW He–Ne laser emitting vertically polarized light of 632.8 nm wavelength.

**Synthesis of MI-functionalized TPE derivative TPE-2MI (Scheme 3).** Zn powder (2.60 g, 40.0 mmol) and 4-aminobenzophenone (3.94 g, 20.0 mmol) were placed into a 250 mL two-necked flask equipped with a condenser. The flask was evacuated under vacuum and flushed with argon three times, followed by the addition of anhydrous THF (60 mL). Then the mixture was cooled to 0 $^oC$, and $TiCl_4$ (2.2 mL, 20.0 mmol) was added dropwise. The mixture was slowly warmed to room temperature, stirred for 30 min, and then refluxed overnight. After cooling down to room temperature, the reaction was quenched by 10% aqueous $K_2CO_3$ solution. After vigorous stirring for 5 min, the dispersed insoluble material was removed by vacuum filtration using a Celite pad. The organic phase was separated, and the aqueous layer was extracted three times with diethyl ether (60 mL × 3). The combined organic fractions were washed with brine and dried over $MgSO_4$. The solvent was removed to give the product 4,4'-(1,2-diphenylethene-1,2-diyl)dianiline (TPE-$2NH_2$, 2.50 g).

A solution of TPE-$2NH_2$ (2.50 g, 6.9 mmol) and maleic anhydride (2.02 g, 20.7 mmol) in 50 mL of THF was stirred at room temperature for 12 h. After solvent evaporation, a yellow solid was obtained, and then directly dissolved in acetic anhydride (50 mL). Then anhydrous sodium acetate (0.24 g, 2.9 mmol) was added, and the resulted mixture was stirred at 75 °C for 6 h. After cooling the solution to 0 °C, a faint yellow solid was precipitated. The precipitate was filtered and washed with hexane to give the TPE-2MI (2.73g, 60% yield in two steps). $^1H$ NMR (500 MHz, $CDCl_3$) $\delta$ 6.83 (s, 4H), 7.07-7.28 (18H, m). $^{13}C$ NMR (125 MHz, $CDCl_3$) $\delta$ 124.8, 126.9, 127.9, 129.5, 131.4, 131.9, 134.2, 140.7, 142.9, 169.4.

**Synthesis of furan-modified random copolymer PMFM, PDFM, or PLFM (Scheme 5).** In a typical procedure for the synthesis of $PM_{62}F_{24}M$, 2-cyano-2-propyl dodecyl trithiocarbonate (24 mg, 0.07 mmol), AIBN (5 mg, 0.03 mmol), furfuryl methacrylate (0.33 g, 2.0 mmol), methyl methacrylate (0.60 g, 6.0 mmol), and DMF (10 mL) was placed into a 25 mL Schlenk flask. The mixture was degassed by three freeze-pump-thaw (FTP) cycles and then was immediately immersed into an oil bath set at 80 $^oC$ to start the polymerization under stirring. After 24 h, the polymerization was stopped by cooling in a liquid nitrogen bath. The cloudy solution was heated to clear and poured into a large amount of cold methanol (~ 500 mL) with stirring. The white solid

was filtered, dried under vacuum, and characterized by $^1$H NMR and GPC ($PM_{62}F_{24}M$, 740 mg, $M_{n, NMR}$ = 10.5×10$^3$, Đ = 1.09).

**Synthesis of cross-linked polymers with TPE-2MI.** A typical procedure for preparing the network PMFM/TPE-2MI with $I_{mal/fur}$ = 1 is given below. Linear copolymer $PM_{60}F_{40}M$ (0.30 g; containing ~ 0.92 mmol furan groups) and TPE-2MI (0.24 g, ~ 0.92 mmol maleimide groups) were co-dissolved in DMF (10 mL) at RT. The obtained homogeneous solution was stirred at 80 $^oC$ for 24 h, resulting in a brown DMF solution. Then the cured $PM_{60}F_{40}M$/TPE-2MI was obtained by removing the DMF under vacuum.

In a typical procedure for preparing the PMFM/TPE-2MI film, linear copolymer $PM_{60}F_{40}M$ (0.30 g; containing ~0.92 mmol furan groups) and TPE-2MI (0.24 g, 0.92 mmol maleimide groups) were dissolved in DCM (10 mL), followed by undergoing spin coating process on glass substrate immediately.

**Preparation of TPE-doped cross-linked polymer PLFM/BMI.** A typical procedure for preparing the TPE-doped network PLFM/BMI with $I_{mal/fur}$ = 0.5 is given below. Linear copolymer $PL_{100}F_{10}M$ (0.50 g; containing ~ 0.18 mmol furan groups), BMI (0.03 g, 0.09 mmol maleimide groups), and TPE (0.03 g, 0.09 mmol) were co-dissolved in DCM (10 mL), followed by immediate removal of the solvent under vacuum and curing at room temperature. At different time intervals, about 5 mg of the bulk sample was taken out for DSC testing.

Part of the previously prepared DCM solution was taken out and immediately underwent a spin coating process on a glass substrate to prepare the polymer film of TPE-doped networks. Then the PL intensity of the film was monitored at different time intervals.

## 2. NMR Spectra

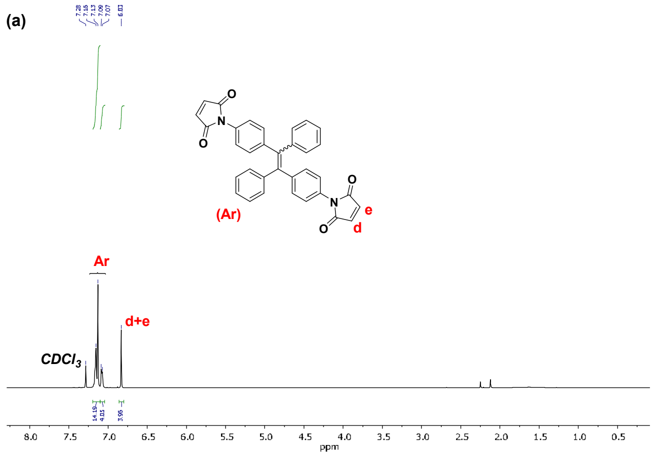


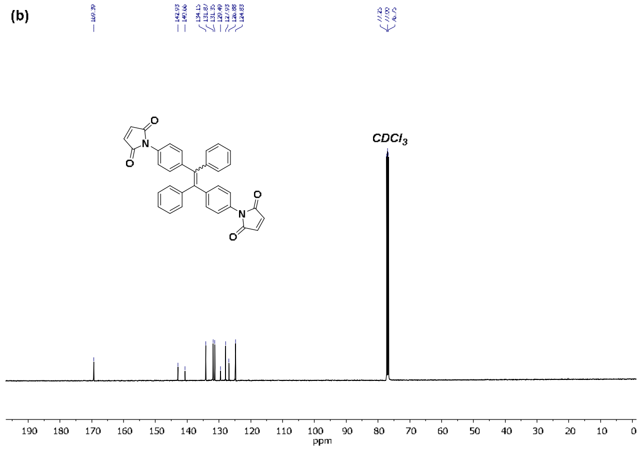


**Figure S1**. $^{1}H$ (a) and $^{13}C$ NMR (b) spectra (400 MHz, $CDCl_3$, 25 °C) of TPE-2MI.

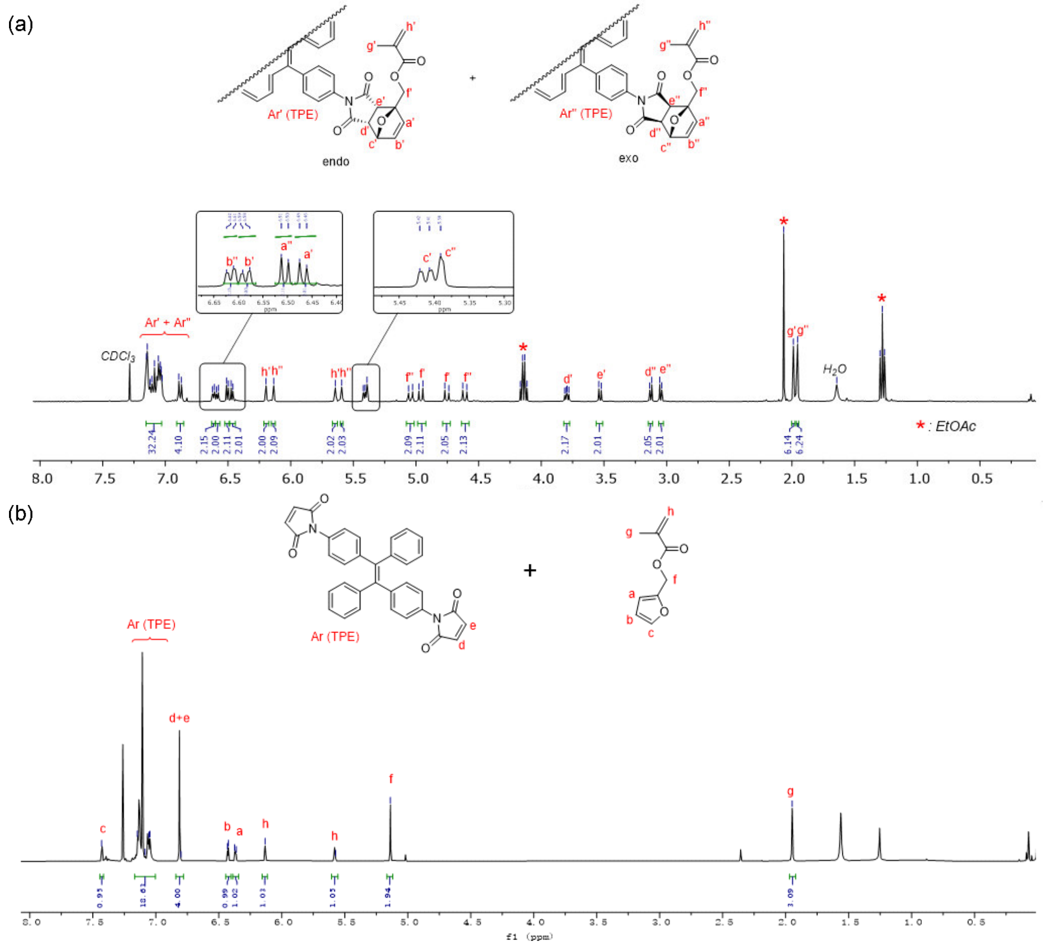


**Figure S2** $^{1}$H NMR spectra (400 MHz, $CDCl_3$, 25 $^{o}$C) of TPE-2AFM (a) and retro-DA reaction product (b).

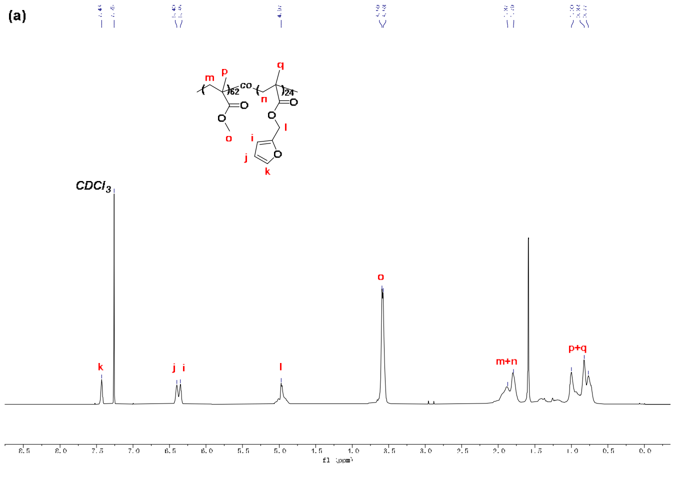

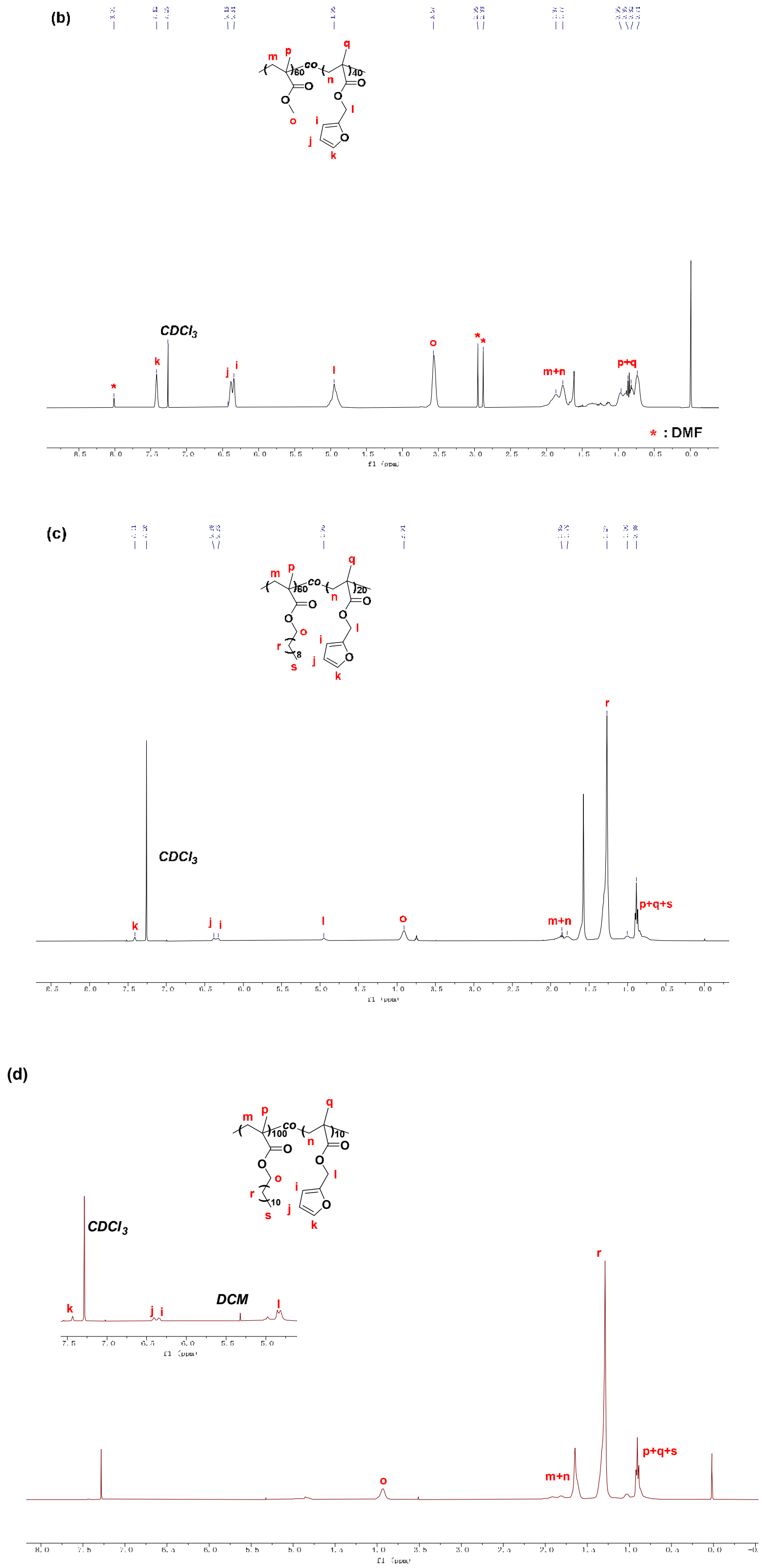


**Figure S3**. $^{1}$H NMR spectra (400 MHz, $CDCl_3$, 25 °C) of $PM_{62}F_{24}M$ (a), $PM_{60}F_{40}M$ (b), $PD_{60}F_{20}M$ (c), and $PL_{100}F_{10}M$ (d).

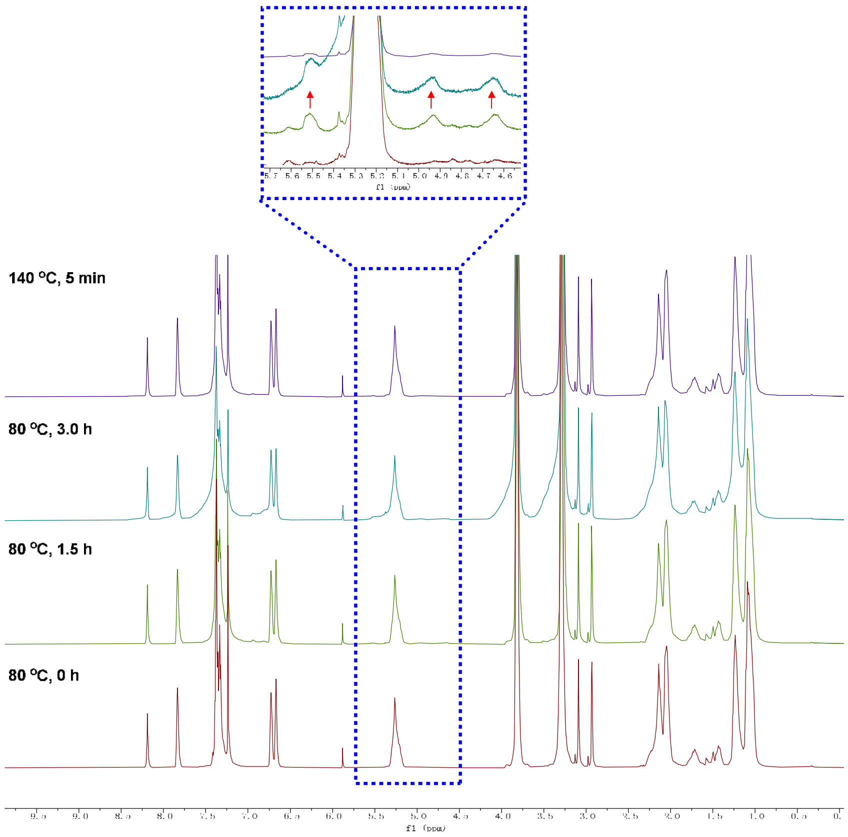


**Figure S4**. $^{1}$H NMR spectra (500 MHz, DMF-$d_7$) of the reaction mixture of $PM_{60}F_{40}M$/TPE-2MI in DMF-$d_7$ after 0 h/1.5 h/3 h heating at 80 °C, and reversal of cross-linked polymer after 5 min heating at 130 °C.

## 3. Photoluminescence (PL) Spectra

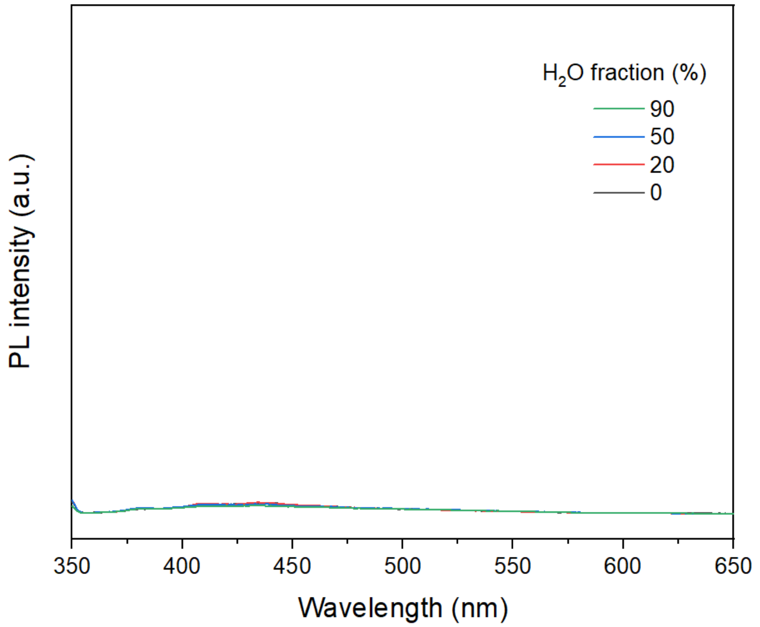


**Figure S5**. PL spectra (Excitation: 342 nm, Temperature: 25 °C) of TPE-2MI in THF/$H_2O$ mixtures at a concentration of 0.1 g/L with different $H_2O$ fractions.

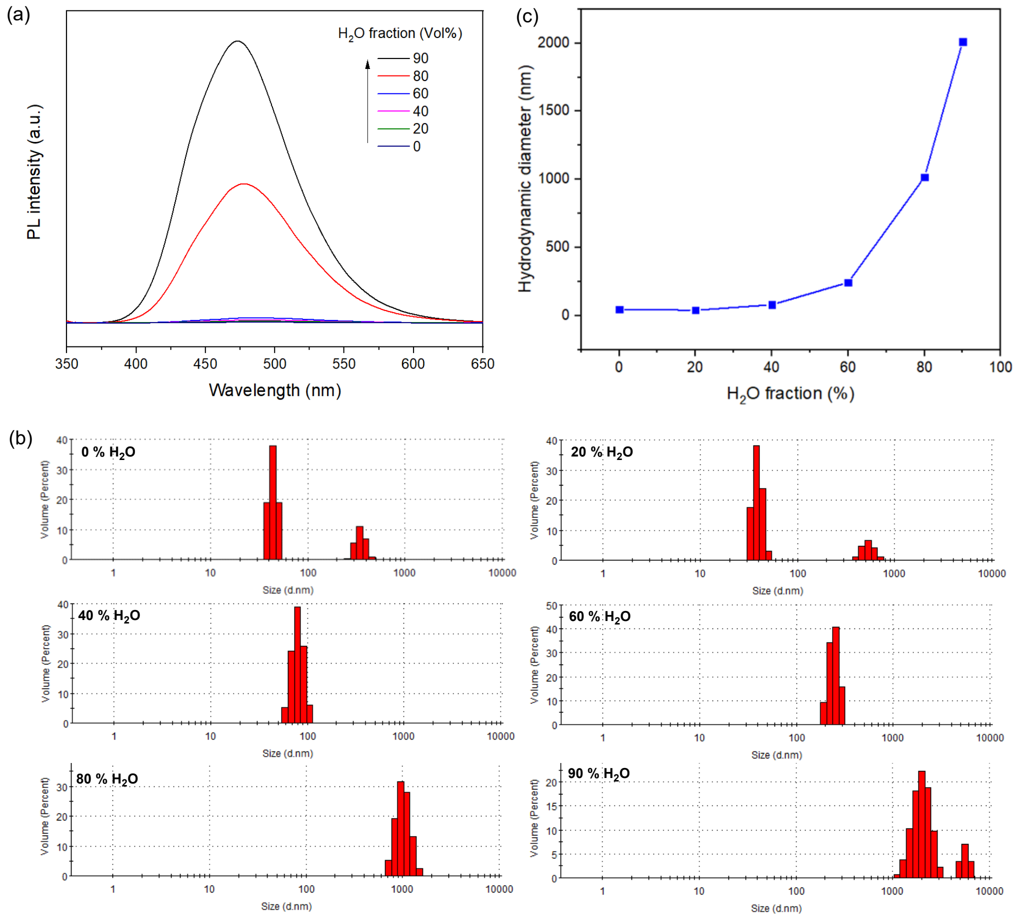


**Figure S6**. (a) PL spectra (Excitation: 342 nm, Temperature: 25 °C) and Size distribution (b) of TPE-2AFM in THF/$H_2O$ mixtures at a concentration of 0.1 g/L with different $H_2O$ fractions; (c) Plot of hydrodynamic diameter of TPE-2AFM in THF/$H_2O$ mixtures as a function of $H_2O$ fraction.

During the temperature-responsive DA/retro-DA reaction, the solution was removed and diluted by adding DMF to monitor PL intensity. When processing cycle 8 was treated, a different batch of commercially available DMF was used, and some unknown impurities were found inside. As shown in Figure S6, a broad peak of about 350-450 nm was found in the PL spectrum of the blind DMF, which is the same as the peak in the repeating cycle 8.

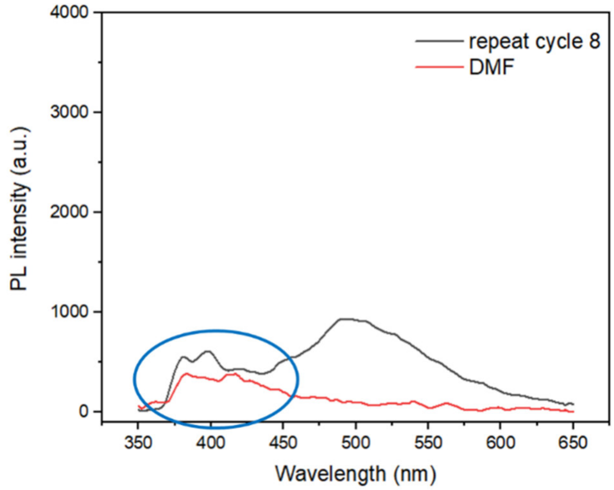


**Figure S7**. PL spectra (Excitation: 342 nm, Temperature: 25 °C) of $PM_{60}F_{40}M$/TPE-2MI networks in DMF solution for repeat cycle 8 (black line) and blind DMF (red line).

As shown in Figure S8, it takes 16 hours to reach equilibrium for the $PM_{62}F_{24}M$/TPE-2MI solution in DMF at 80 °C, while only 4 hours for $PD_{60}F_{20}M$ / TPE-2MI. The long side-chain prevents $PD_{60}F_{20}M$/TPE-2MI networks from further interconnecting to reach equilibrium sooner.

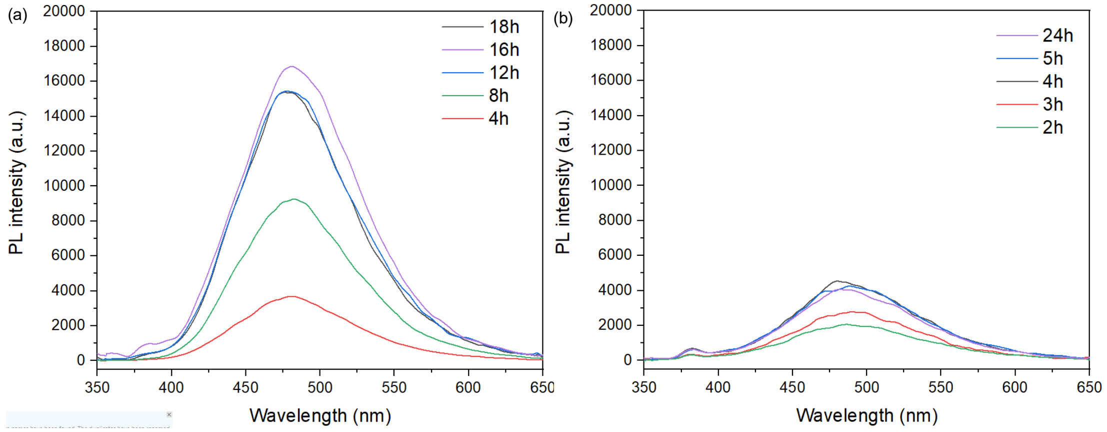


**Figure S8**. PL spectra (Excitation: 342 nm, Temperature: 25 °C) of mixture of $PM_{62}F_{24}M$/TPE-2MI (a) and $PD_{60}F_{20}M$/TPE-2MI (b) in DMF (concentration: 0.8 g/L) at different time intervals.

$PL_{100}F_{10}M$, and TPE-2MI ($I_{mal/fur}$ = 1 or 0.5) were dissolved in dichloromethane, followed by a spin coating process on a glass substrate immediately. Then the PL intensity of the polymer film was monitored at different times. The PL spectra are shown in Figure S9.

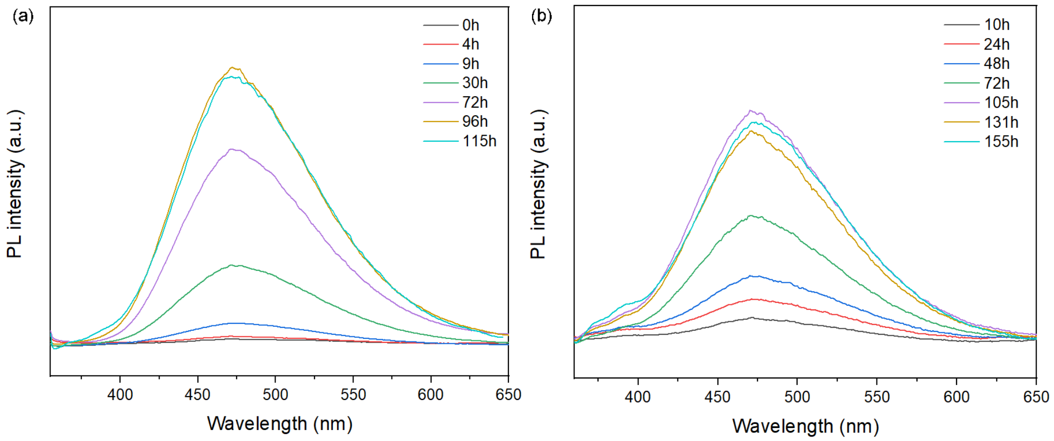


**Figure S9**. PL spectra (Excitation: 342 nm, Temperature: 25 °C) of polymer film of $PL_{100}F_{10}M$/TPE-2MI with $I_{mal/fur}$ = 1 (a) or 0.5 (b) at different time intervals.

$PL_{100}F_{10}M$, BMI, and TPE ($I_{mal/fur}$ = 0.5) were dissolved in dichloromethane, followed by a spin coating process on a glass substrate immediately. Then the PL intensity of the polymer film was monitored at different times. The PL spectra are shown in Figure S10.

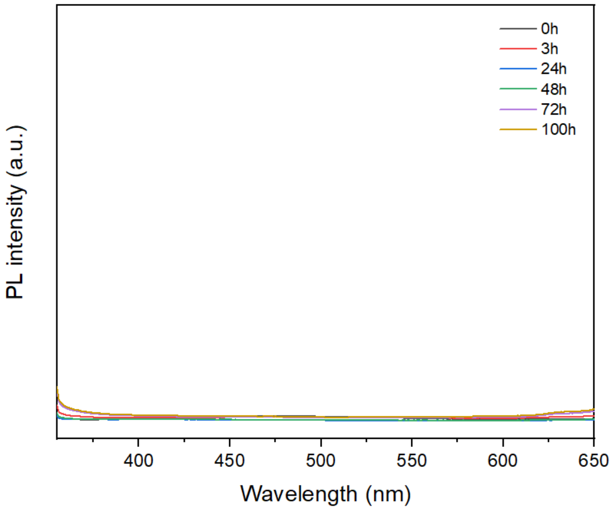


**Figure S10**. PL spectra (Excitation: 342 nm, Temperature: 25 °C) of the film of TPE-doped networks $PL_{100}F_{10}M$/BMI ($I_{mal/fur}$ = 0.5) at different time intervals.

## 4. GPC Traces

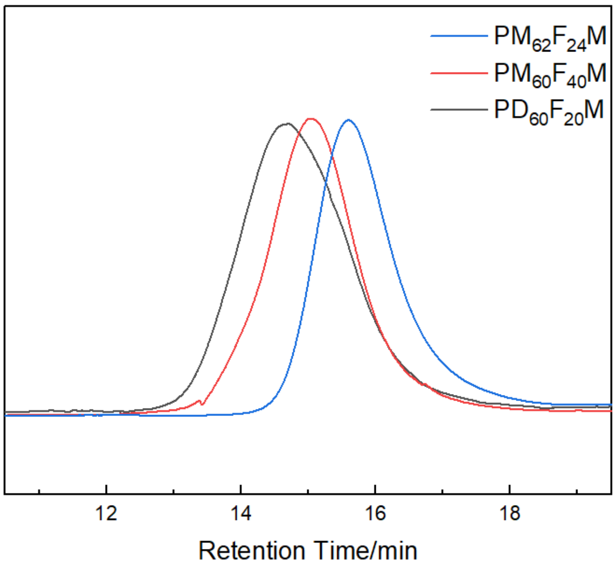


**Figure S11**. GPC (THF, 25 °C, PS standards) traces of different random copolymers PMFM and PDFM.

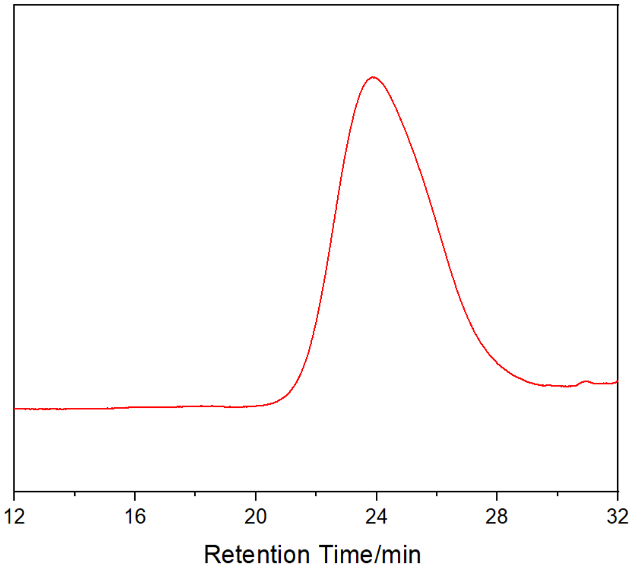


**Figure S12**. GPC (THF, 25 °C, PS standards) trace of random copolymer $PL_{100}F_{10}M$.

## 5. DSC Thermograms

All the samples were heated to 50 °C to erase thermal history, cooled to -60 °C (or -80 °C in some cases) at 10 °C/min, and then heated to 150 °C at 10 °C/min. Specific samples were heated to a higher temperature, e.g., 175 °C, to examine the DA and retro-DA reactions during the heating process. Glass transition temperatures ($T_g$) were obtained from the second heating ramp.

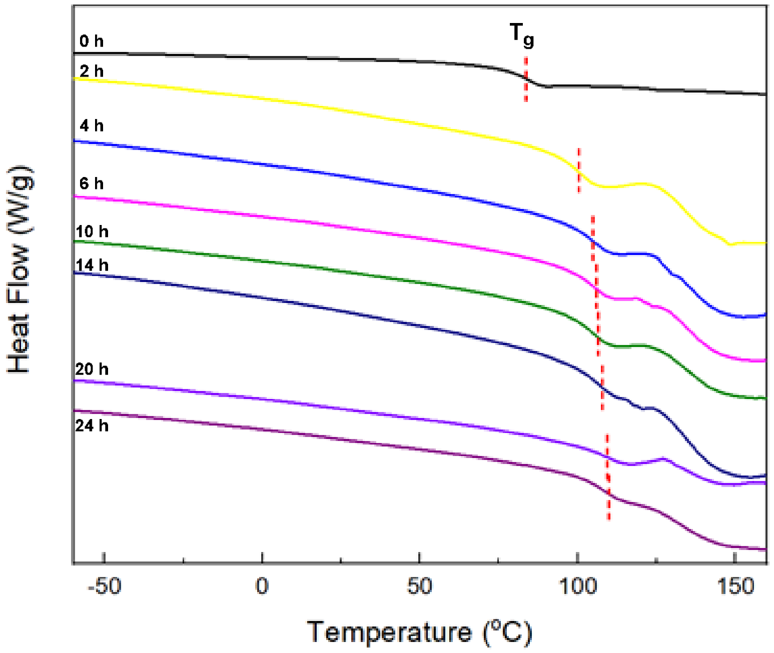


**Figure S13**. DSC thermograms for $PM_{60}F_{40}M$/TPE-2MI polymer networks obtained from its DMF solution at different time intervals.

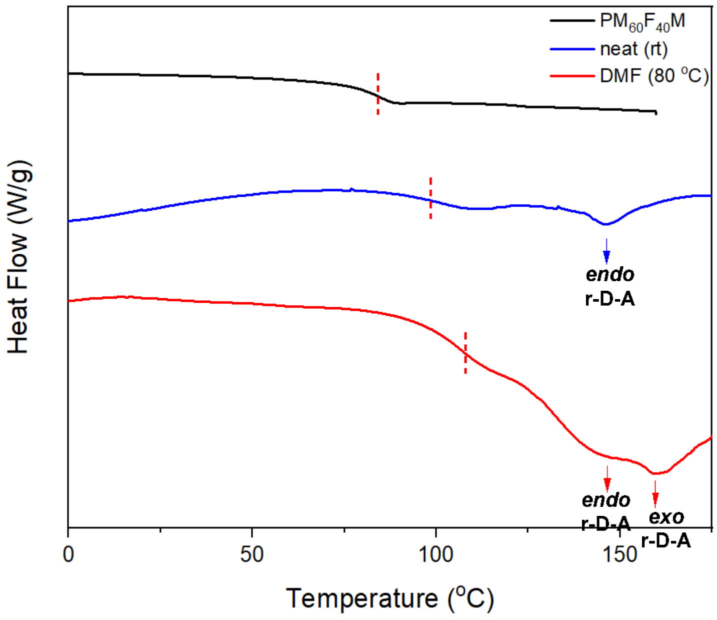


**Figure S14**. DSC thermograms for $PM_{60}F_{40}M$ (black line), $PM_{60}F_{40}M$/TPE-2MI cross-linked networks (neat for 1 week, blue line), and $PM_{60}F_{40}M$/TPE-2MI cross-linked networks (DMF solution for 24 hours, red line).

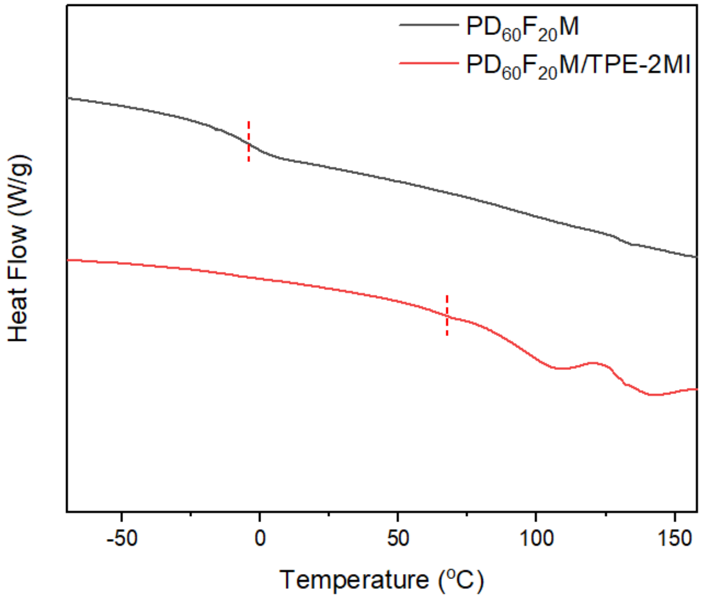


**Figure S15**. DSC thermograms for $PD_{60}F_{20}M$ (black line) and $PD_{60}F_{20}M$/TPE-2MI cross-linked networks (red line, DMF solution at 80 °C for 24 hours).

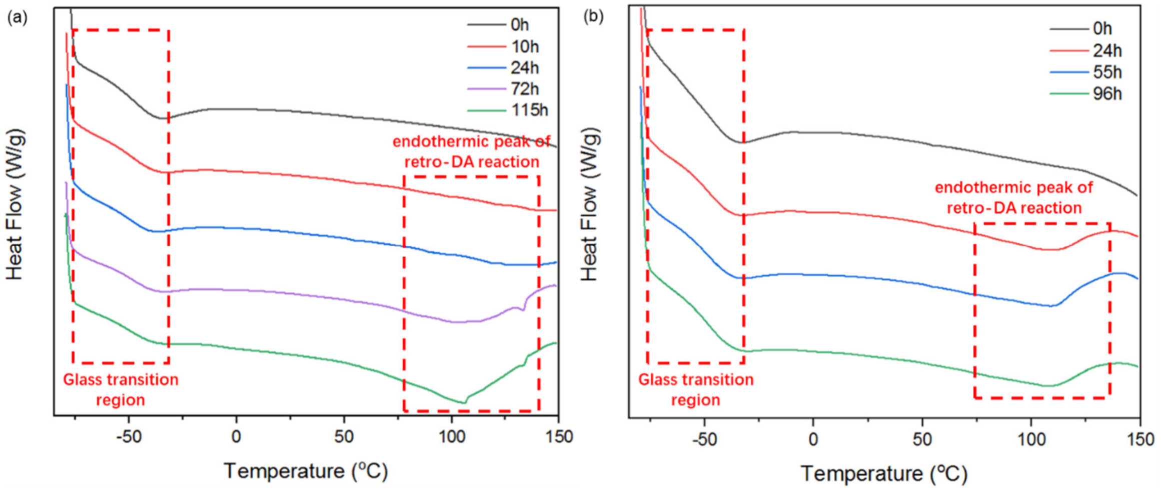


**Figure S16**. DSC thermograms for bulk $PL_{100}F_{10}M$/TPE-2MI with $I_{mal/fur}$ = 1 (a) or 0.5 (b) at different time intervals.

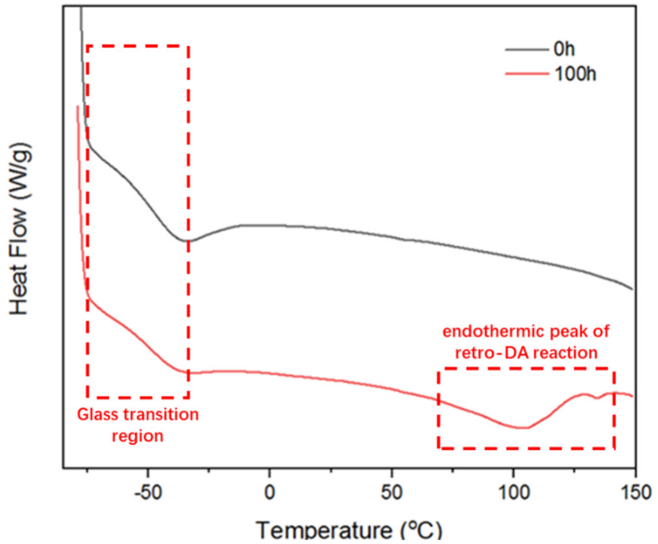


**Figure S17**. DSC thermograms for bulk $PL_{100}F_{10}M$/BMI/TPE ($I_{mal/fur}$ = 0.5) at different time intervals.

## 6. Pictures

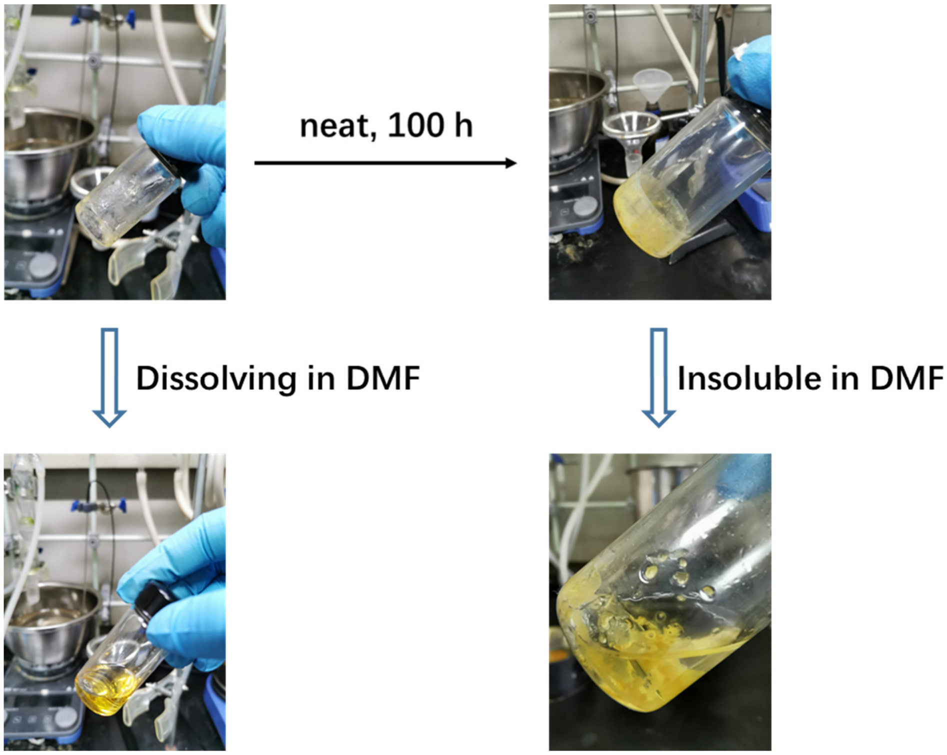


**Figure S18**. The bulk $PL_{100}F_{10}M$/TPE-2MI mixture ($I_{mal/fur}$ = 0.5) becomes insoluble in DMF after cross-linking at room temperature.

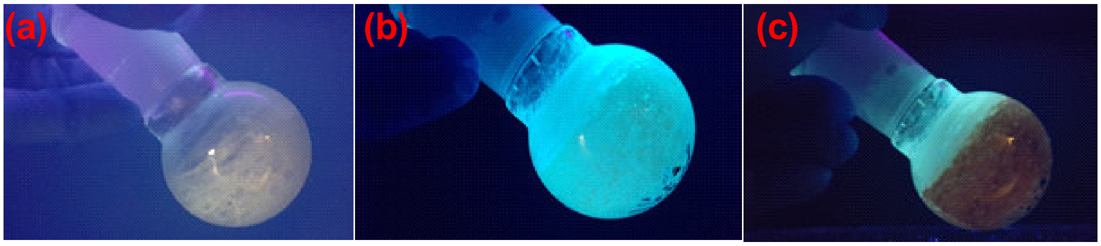


**Figure S19**. Bulk $PM_{60}F_{40}M$/TPE-2MI mixture cured at room temperature for 0 h (a), 20 h (b), and then put in an oil bath (140 $^{o}$C) for 10 min (c). All the pictures were taken under 365 nm UV light.
